# Supplementary material for: Simulating hyperbolic space on a circuit board
Source: Nat Commun. 2022 Jul 28;13:4373. doi: 10.1038/s41467-022-32042-4 (PMC9334621; doi:10.1038/s41467-022-32042-4)
Supplement: Supplementary file 1 — Supplementary Information File [file 41467_2022_32042_MOESM1_ESM.pdf]

Supplementary Information to:
Simulating hyperbolic space on a circuit board

Patrick M. Lenggenhager [1,2,3,*] Alexander Stegmaier [4,*] Lavi K. Upreti [4] Tobias Hofmann [4]
Tobias Helbig [4] Achim Vollhardt [2] Martin Greiter,[4] Ching Hua Lee,[5] Stefan Imhof,[6] Hauke Brand [6]
Tobias Kießling,[6] Igor Boettcher [7,8] Titus Neupert [2,†] Ronny Thomale [4,†] and Tomáš Bzdušek [1,2,†]

[1] *Condensed Matter Theory Group, Paul Scherrer Institute, 5232 Villigen PSI, Switzerland*
[2] *Department of Physics, University of Zurich, Winterthurerstrasse 190, 8057 Zurich, Switzerland*
[3] *Institute for Theoretical Physics, ETH Zurich, 8093 Zurich, Switzerland*
[4] *Institut für Theoretische Physik und Astrophysik, Universität Würzburg, 97074 Würzburg, Germany*
[5] *Department of Physics, National University of Singapore, Singapore 117551, Republic of Singapore*
[6] *Physikalisches Institut, Universität Würzburg, 97074 Würzburg, Germany*
[7] *Department of Physics, University of Alberta, Edmonton, Alberta T6G 2E1, Canada*
[8] *Theoretical Physics Institute, University of Alberta, Edmonton, Alberta T6G 2E1, Canada*

List of Supplementary Notes

1. Eigenmodes of the Laplace-Beltrami operator 1
   a. Euclidean space 2
   b. Hyperbolic space 2

2. Lattice regularization of the graph Laplacian 3
   a. Euclidean space 3
   b. Hyperbolic space 4
   c. Approximating the continuum 4

3. Comparison of tessellations of hyperbolic space 5
   a. Approximating the continuum 6
   b. Signatures of negative curvature 7
   c. Role of rotation symmetry 7

4. Parasitic resistances 8

5. Extended Analysis of Measured Eigenmodes 9

6. Signal propagation in the electric circuit network 12

SUPPLEMENTARY NOTE 1. EIGENMODES OF THE LAPLACE-BELTRAMI OPERATOR

The general Laplace-Beltrami operator for a given metric tensor g_{ij} is

$$\Delta_g = \frac{1}{\sqrt{\det(g)}}\partial_i\left(\sqrt{\det(g)}g^{ij}\partial_j\right), \tag{1}$$

where g^{ij} is the matrix inverse of g_{ij}. In Euclidean space, we have $(g_{\mathrm{E}})_{ij} = \delta_{ij}$, such that $\Delta_{\mathrm{E}} = \partial_x^2 + \partial_y^2$, the usual Laplace operator. In contrast, for the Poincaré disk representation of the hyperbolic plane ($\mathcal{D} = \{(x,y) \in \mathbb{R}^2 \,|\, x^2+y^2 = r^2 < 1\}$ with length element $\mathrm{d}s^2 = (1-x^2-y^2)^{-2}(dx^2+dy^2)$ corresponding to constant negative curvature $K=-4$),

$$(g_{\mathrm{H}})_{ij} = (1-r^2)^{-2}\delta_{ij}, \tag{2}$$

such that the Laplace-Beltrami operator is

$$\Delta_{\mathrm{H}} = \left(1-\left(x^2+y^2\right)\right)^2\left(\partial_x^2+\partial_y^2\right). \tag{3}$$

We now consider the disk $\mathcal{D}_{r_0} := \{(x,y) \in \mathbb{R}^2 \,|x^2+y^2 \leq r_0^2\} \subset \mathcal{D}$ and rewrite the Laplace-Beltrami operator in polar coordinates $x = r\cos(\theta)$, $y = r\sin(\theta)$:

$$\Delta_{\mathrm{E}} = \partial_r^2 + \frac{1}{r}\partial_r + \frac{1}{r^2}\partial_\theta^2, \tag{4}$$

$$\Delta_{\mathrm{H}} = \left(1-r^2\right)^2 \left(\partial_r^2 + \frac{1}{r}\partial_r + \frac{1}{r^2}\partial_\theta^2\right). \tag{5}$$

We are interested in eigenmodes of $-\Delta_{\mathrm{g}}$, where $\mathrm{g} \in \{\mathrm{E}, \mathrm{H}\}$ indicates the geometry, i.e. solutions to the Dirichlet problem

$$(\Delta_{\mathrm{g}} + \lambda)u(x,y) = 0, \qquad u(x,y)|_{(x,y)\in\partial\mathcal{D}_{r_0}} = 0. \tag{6}$$

a. Euclidean space

We first discuss the solutions to Supplementary Equation (6) in the Euclidean case. The differential equation is separable, such that we can make the ansatz $u(x,y) = R(r)\Theta(\theta)$ and find

$$-\frac{\Theta''(\theta)}{\Theta(\theta)} = \frac{r^2R''(r) + rR'(r) + r^2\lambda R(r)}{R(r)}. \tag{7}$$

Since, we are on the disk, $\Theta(\theta + 2\pi) = \Theta(\theta)$, such that $\Theta(\theta) = \mathrm{e}^{\mathrm{i}\ell\theta}$ for $\ell \in \mathbb{Z}$ and

$$r^2R''(r) + rR'(r) + (k^2r^2 - \ell^2)R(r) = 0, \tag{8}$$

where we substituted $\lambda = k^2$. With the further substitution $\rho = kr$, we obtain

$$\rho^2R''(\rho) + \rho R'(\rho) + (\rho^2 - \ell^2)R(\rho) = 0, \tag{9}$$

which is the Bessel equation, such that the solutions are given by the Bessel functions of the first kind

$$u_{\mathrm{E}}^{n\ell}(x,y) = \mathcal{J}_\ell(k_n r)\mathrm{e}^{\mathrm{i}\ell\theta} \tag{10}$$

where $k_n = z_n/r_0$ and z_n is the n-th root of $\mathcal{J}_\ell$.

b. Hyperbolic space

We proceed analogously in the hyperbolic case, where the same ansatz $u(x,y) = R(r)\Theta(\theta)$ results in

$$(1-r^2)^2r^2R''(r) + (1-r^2)^2rR'(r) + (\lambda r^2 - \ell^2(1-r^2)^2)R(r) = 0. \tag{11}$$

Introducing $s := (1+r^2)/(1-r^2)$, this can be rewritten as

$$2sR'(s) - 4r^2(1-s^2)R''(s) + (\lambda r^2 - \ell^2(1-r^2)^2)R(s) = 0 \tag{12}$$

Dividing by $-4r^2$ and setting $\lambda = -4q(q+1) = 1 + k^2$, we find

$$\left((1-s^2)\partial_s^2 - 2s\partial_s + \left(q(q+1) - \ell^2\frac{1}{1-s^2}\right)\right)R(s) = 0, \tag{13}$$

whose solutions are the associated Legendre functions $P_q^\ell(s)$, such that we obtain

$$u_{n\ell}(x,y) = P^\ell_{\frac{1}{2}(-1+\mathrm{i}k_{n\ell})}\left(\frac{1+r^2}{1-r^2}\right)\mathrm{e}^{\mathrm{i}\ell\theta} \tag{14}$$

with $k_{n\ell}$ being the n-th root of

$$k \mapsto P^\ell_{\frac{1}{2}(-1+\mathrm{i}k)}\left(\frac{1+r_0^2}{1-r_0^2}\right) \tag{15}$$

and $\ell \in \mathbb{Z}$ as in the Euclidean case.

SUPPLEMENTARY NOTE 2. LATTICE REGULARIZATION OF THE GRAPH LAPLACIAN

As discussed in the Methods, the graph Laplacian can be approximated by the continuum Laplace-Beltrami operator in the leading order in the distance between lattice sites, see e.g., Eqs. (8) and (9) in Methods. In Supplementary Notes 2 a and 2 b we present a detailed derivation of this expansion for regular tessellations with equivalent sites where all distances between adjacent sites are equal (in the corresponding metric). Such tessellations are called Archimedian. They are generally denoted by their vertex configuration $n_1.n_2.\cdots.n_q$, where $n_1, n_2, \ldots, n_q$ give the number of sides of the q regular polygons meeting at each vertex. The tessellations considered in the main text are a special case called Platonic tessellations, because they have q copies of the same regular p-gon meeting at each vertex. In Supplementary Note 2 c we briefly discuss how to quantify how well a certain tessellation approximates the continuum.

We now consider Archimedian tessellations of the unit disk for both Euclidean and hyperbolic space (in the Poincaré disk representation) with Dirichlet boundary conditions imposed. For convenience, we parametrize the coordinates (x, y) of the Euclidean plane and the Poincaré disk using complex numbers $z := x + \mathrm{i}y$, where z lies in the infinite complex plane for the Euclidean and in the complex unit disk for the hyperbolic case. The boundary condition implies that all vertices, including those on the non-vanishing boundary, are equivalent. Recall that the graph Laplacian is a matrix $Q = A - D$ with entries Q_{ab} and any test function $u(z)$ on the complex unit disk induces a function on the lattice, via $a \mapsto u(z_a) = u_a$. We closely follow Appendix B of Ref. 1 to express the action of Q on u_a in terms of the Laplace-Beltrami operator. The action of the graph Laplacian Q on the test function $a \mapsto u_a$ at an arbitrary site a (using Einstein's summation convention) is then

$$Q_{ab}u_b = A_{ab}u_b - D_{ab}u_b = \sum_{i=1}^{q} u(z_{a+e_k}) - qu(z_a), \tag{16}$$

where z_{a+e_i} denote the position of the sites adjacent to site a.

a. Euclidean space

We first discuss a Euclidean tessellation with coordination q, i.e., where each site has q adjacent sites. Let d be the distance between two adjacent sites, then

$$z_{a+e_i} = z_a + d\mathrm{e}^{\mathrm{i}\phi_a}\mathrm{e}^{\mathrm{i}\frac{2\pi}{q}(i-1)} =: z_a + dw_{ai}, \tag{17}$$

where ϕ_a is a site-dependent phase factor, and we can expand $u(z_{a+e_i})$ in powers of d:

$$\begin{aligned} u(z_{a+e_i}) &= u(z_a) + \frac{\mathrm{d}}{\mathrm{d}d}u(z_{a+e_i})\Big|_{d=0} d + \frac{1}{2}\frac{\mathrm{d}^2}{\mathrm{d}d^2}u(z_{a+e_i})\Big|_{d=0} d^2 + \mathcal{O}\big(d^3\big) \\ &= u(z_a) + \big(w_{ai}\partial_z + \bar{w}_{ai}\bar{\partial}_z\big)\, u(z)\big|_{z=z_a} d + \frac{1}{2}\big(w_{ai}\partial_z + \bar{w}_{ai}\bar{\partial}_z\big)^2 u(z)\Big|_{z=z_a} d^2 + \mathcal{O}\big(d^3\big) \end{aligned} \tag{18}$$

with $\partial_z = \partial/\partial_z$ and $\bar{\partial}_z = \partial/\partial_{\bar{z}}$ and $\bar{\cdot}$ denoting complex conjugation. Note that for any $m \in \mathbb{Z}$

$$\sum_{i=1}^{q} w_{ai}^m = \mathrm{e}^{\mathrm{i}\phi_a}\sum_{i=1}^{q}\mathrm{e}^{\mathrm{i}\frac{2\pi m}{q}(i-1)} = 0, \tag{19}$$

and $|w_{ai}| = 1$, such that ϕ_a drops from the subsequent calculations:

$$\sum_{i=1}^{q} u(z_{a+e_a}) = qu(z_a) + qd^2\, \partial_z\bar{\partial}_z u(z)\big|_{z=z_a} + \mathcal{O}\big(d^3\big). \tag{20}$$

Since $\Delta_{\mathrm{E}} = 4\partial_z\bar{\partial}_z$, we finally find

$$Q_{ab}u_b = \frac{q}{4}d^2\Delta_{\mathrm{E}}u(z_a) + \mathcal{O}\big(d^3\big). \tag{21}$$

With $q = 6$ for a $\{3, 6\}$ tessellation this reproduces Eq. (8) in Methods.

b. Hyperbolic space

We proceed analogously for hyperbolic tessellations with coordination q and hyperbolic distance d_0 between adjacent sites. Here it is helpful to first transform the Poincaré disk by the automorphism

$$z \mapsto v(z) = \frac{z_a - z}{1 - z\bar{z}_a}. \tag{22}$$

This transformation corresponds to a π-rotation that exchanges z_a and the origin. In particular, note that it squares to identity, implying that $z \mapsto v(z)$ and its inverse $z \mapsto v^{-1}(z)$ are equivalent. Recall further that the hyperbolic distance between the origin and an arbitrary point z in the unit disk takes the form $d = \operatorname{arctanh}(|z|)$. In the transformed coordinates, z_{a+e_i} takes the simple form

$$v_{a+e_i} = v(z_{a+e_i}) = h\mathrm{e}^{\mathrm{i}\phi_a}\mathrm{e}^{\mathrm{i}\frac{2\pi}{q}(i-1)} = hw_{ai} \tag{23}$$

with $h = \tanh(d_0)$, $u(z_{a+e_i}) = u(z(v_{a+e_i}))$, and ϕ_a being again a site-dependent phase factor that subsequently drops out from the calculations. Expanding in powers of h, we obtain

$$\begin{aligned} u(z_{a+e_i}) &= u(z_a) + \frac{\mathrm{d}}{\mathrm{d}h}u(z(v_{a+e_i}))\bigg|_{h=0} h + \frac{1}{2}\frac{\mathrm{d}^2}{\mathrm{d}h^2}u(z(v_{a+e_i}))\bigg|_{h=0} h^2 + \mathcal{O}(h^3) \\ &= u(z_a) - (1-|z_a|^2)\left(w_{ai}\partial_z + \bar{w}_{ai}\bar{\partial}_z\right)u(z)\big|_{z=z_a} h + \frac{1}{2}(1-|z_a|^2)^2\left(w_{ai}\partial_z + \bar{w}_{ai}\bar{\partial}_z\right)^2 u(z)\Big|_{z=z_a} h^2 + \mathcal{O}(h^3) \end{aligned} \tag{24}$$

Since w_{ai} are still the same as in the Euclidean case, Supplementary Equation (16) becomes

$$Q_{ab}u_b = \frac{q}{4}h^2\,(1-|z|^2)^2\Delta_{\mathrm{E}}u(z)\Big|_{z=z_a} + \mathcal{O}(h^3) \tag{25}$$

and recalling that $(1-|z|^2)^2\Delta_{\mathrm{E}} = \Delta_{\mathrm{H}}$, we finally arrive at

$$Q_{ab}u_b = \frac{q}{4}h^2\Delta_{\mathrm{H}}u(z_a) + \mathcal{O}(h^3). \tag{26}$$

With $q = 7$ for a $\{3,7\}$ tessellation this reproduces Eq. (9) in Methods.

c. Approximating the continuum

How faithfully a given tessellation approximates the continuum with respect to the Laplace-Beltrami operator can be quantified according to several different aspects. Recall that, according to Supplementary Equation (25), the graph Laplacian can be interpreted as the leading-order term of an expansion in $h = \tanh(d_0)$ (where d_0 is the hyperbolic distance between neighboring sites) of the Laplace-Beltrami operator. While this allows us to compare different tessellations, it does not directly quantify how good the approximation is for any particular tessellation. To perform such a quantitative assessment, certain properties can be computed both on a continuous disk as well as on the lattice and then compared to each other.

For example, in Ref. 1 the authors compute the ground state energy and Green function of the Hamiltonian given by $-A$, where A is the adjacency matrix of the graph induced by the tessellation. In the main text we have compared the ordering of the eigenmodes of the Laplace-Beltrami operator (with appropriate boundary conditions) according to increasing eigenvalues to the one of the graph Laplacian, see also Supplementary Figure 1a. To formulate a more quantitative criterion, we consider the eigenmodes directly, computing the overlap of the eigenvectors of the graph Laplacian with the discretized eigenmodes of the Laplace-Beltrami operator, shown in Supplementary Figure 1b. In addition, the Laplacian eigenvalues can also be quantitatively compared; we do the latter in Supplementary Note 3 a, when comparing different tessellations.

For both comparisons, the first step is to match the eigenvectors to appropriate eigenmodes. This is achieved by first computing the overlap of a given eigenvector of the graph Laplacian with the 85 eigenmodes of the Laplace-Beltrami operator with lowest eigenvalue, and by subsequently determining the quantum numbers n and $|\ell|$ (see Supplementary Note 1) of the modes with largest overlap (see inset of Supplementary Figure 1b). Here, by overlap we mean the dot product of normalized eigenmodes for the graph vs. continuum Laplacian. We observe in Supplementary Figure 1b that the maximal overlap is very close to 1 up to (and excluding) mode 17, which corresponds to the first mode whose order does not agree with the continuum case anymore: mode 17 in the continuum is the one with $(n, |\ell|) = (1, 1)$, while on the lattice it is $(0, 8)$. For the mode reordering to be observable, the overlap has to be close to 1 for all the modes up to (and including) the $(1, 0)$ mode, which in our case is mode 10. Note that due to the small number of lattice sites, the quantitative agreement of the eigenvalues is not particularly good, but is is improved significantly when increasing the number of sites, cf. Supplementary Figure 2. However, for our purposes the overlap of the eigenmodes is sufficient to guarantee the reordering.

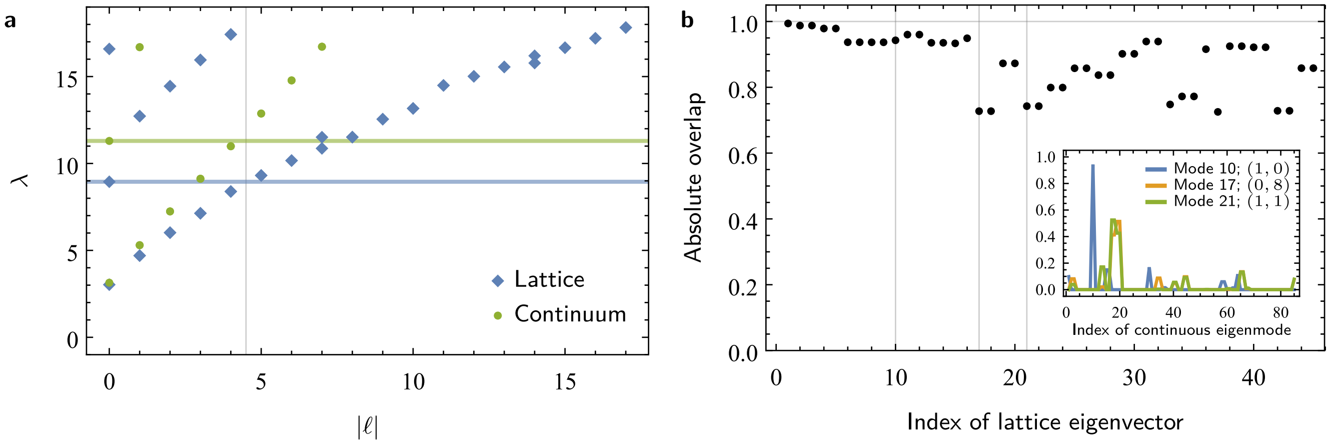

Supplementary Figure 1. **Comparison between lattice and continuum.** **a** Eigenvalues λ of the Laplace-Beltrami operator (green disks) with Dirichlet boundary conditions at $r_0 = 0.94$ and the graph Laplacian (blue diamonds) of the graph obtained from the $\{3, 7\}$ tessellation with 85 sites as a function of the absolute value of the angular momentum quantum number ℓ. Horizontal lines indicate the value of λ for the $(1, 0)$ mode emphasizing the reordering of the modes compared to flat space (see Fig. **2** in the main text). **b** Absolute value of the overlap of the eigenvectors of the graph Laplacian (with index according to increasing eigenvalue given on the horizontal axis) with the discretized eigenmodes of the Laplace-Beltrami operator for the first 45 eigenvectors. The inset shows examples for the overlap of three eigenvectors (indicated by vertical gray lines in the main plot) with the first 85 eigenmodes of the Laplace-Beltrami operator. For each, the maximum overlap is identified and from the corresponding eigenmode n and ℓ are extracted (see legend). Note that in the panel the total overlap of the graph Laplacian's eigenvectors with the corresponding eigenmodes $(n, \pm |\ell|)$ is given (where the total is defined as the square root of the sum of squares of the individual overlaps), while in the inset the overlap with $(n, + |\ell|)$ and $(n, + |\ell|)$ is given separately.

SUPPLEMENTARY NOTE 3. COMPARISON OF TESSELLATIONS OF HYPERBOLIC SPACE

In Supplementary Note 2 we have derived that corrections of the graph Laplacian to the continuum Laplace-Beltrami operator are of third order in $h = \tanh(d_0)$ with d_0 being the hyperbolic distance between adjacent lattice sites. This agrees with the intuition that the density of the tessellation determines the accuracy of the approximation of the continuum. This fact should not be misunderstood, however, as implying that the different tessellations differ only in the positions of the sites. On the contrary, different Archimedean tessellations, each specified by $n_1.n_2.\cdots.n_q$, where q is the number of polygons joining at each site and n_i the number of sides of the ith polygon, differ even if viewed as graphs.

For any planar graph, we can define the Euler characteristic per vertex

$$\Delta\chi = \Delta V - \Delta E + \Delta F, \tag{27}$$

where $\Delta V = 1$ is the number of vertices per vertex, ΔE, the number of edges per vertex, and ΔF the number of faces per vertex. The graph induced by the Archimedean tessellation $n_1.n_2.\cdots.n_q$ therefore has Euler characteristic per vertex

$$\Delta\chi = 1 - \frac{q}{2} + \sum_{i=1}^{q} \frac{1}{n_i} = \frac{1}{2}\left(2 - \sum_{i=1}^{q} \frac{n_i - 2}{n_i}\right). \tag{28}$$

As an example, let us compare the Euclidean $\{3, 6\}$ to the hyperbolic $\{3, 7\}$ tessellation. For the former, Supplementary Equation (28) gives $\Delta\chi = 0$, consistent with flat space, and for the latter, $\Delta\chi = -1/6 < 0$, consistent with hyperbolic space.

The Euler characteristic per site allows us, via the Gauss-Bonnet theorem, to compute the area per vertex α. The Gauss-Bonnet theorem relates the Euler characteristic to the curvature

$$\int_\alpha K \,\mathrm{d}A = 2\pi\Delta\chi. \tag{29}$$

In the hyperbolic plane we consider, we have constant curvature $K = -4$, such that the left-hand side evaluates to -4α and we find

$$\alpha = -\frac{\pi}{4}\left(2 - \sum_{i=1}^{q} \frac{n_i - 2}{n_i}\right). \tag{30}$$

In this section we study three hyperbolic tessellations of the hyperbolic plane: (i) $\{3, 7\}$, (ii) $\{7, 3\}$, and (iii) 6.6.7 (also called the hyperbolic soccerball), illustrated in panels a–c of Supplementary Figure 2. The area per vertex for those is (i) $\pi/12$, (ii)

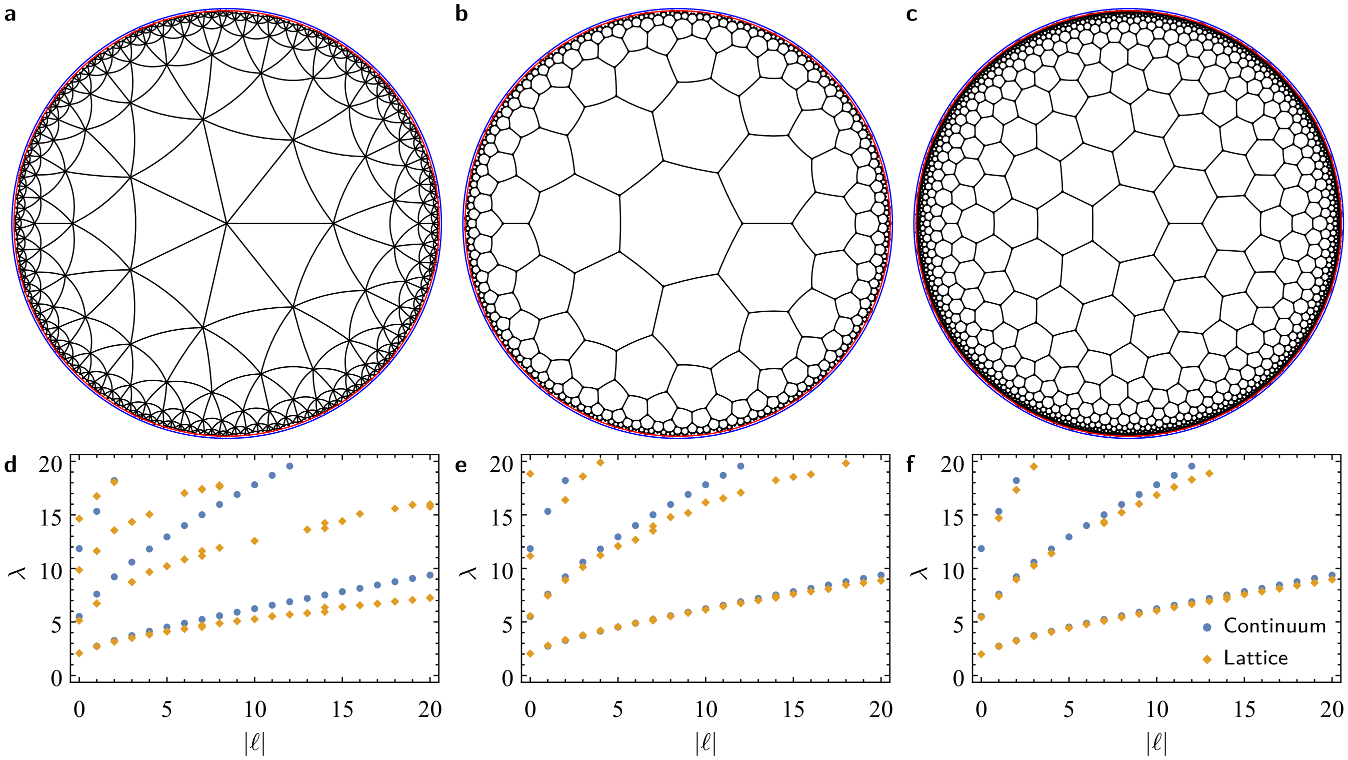

Supplementary Figure 2. **Comparison of hyperbolic tessellations for fixed disk radius. a,b,c** Tessellations $\{3,7\}$, $\{7,3\}$ and $6.6.7$ of the hyperbolic plane in Poincaré disk representation (the unit circle is shown in blue), respectively, covering a disk of radius $r_0 = 0.99$ (red circle). This results in 589, 1197, and 3857 vertices, respectively. **d,e,f** Angular momentum dispersion of the eigenstates of the graph Laplacian for the three tessellations (orange diamonds) compared to the same for the eigenmodes of the continuum Laplace-Beltrami operator on the hyperbolic drum with the same radius r_0 (blue disks).

$\pi/28$, and (iii) $\pi/84$. We compare these three tessellations with respect to three properties: First, in Supplementary Note 3 a we consider the accuracy of approximating the continuum. This is determined by the density of the tessellation, which in turn depends on the vertex configuration (or equivalently on α). Subsequently, in Supplementary Note 3 b, we examine the total (i.e., integrated over the area) curvature that can be obtained in a finite lattice with a fixed number of vertices. Finally, in Supplementary Note 3 c, we discuss the effect of rotation symmetry with respect to a central vertex on the spectrum and the profile of the eigenmodes.

a. Approximating the continuum

Here, we analyze how well the three tessellations shown in Supplementary Figure **2** approximate the continuum by comparing the spectra of the graph Laplacian on the lattice to the ones of the Laplace-Beltrami operator on a corresponding disk. All three tessellations cover approximately the same disk of radius $r_0 < 1$; however, due to them having different area per vertex α, cf. Supplementary Equation (30), the number of vertices varies between the three cases. More specifically, for each of the three tessellations we compare the spectrum of the graph Laplacian Q to the spectrum of the Laplace-Beltrami operator with Dirichlet boundary conditions for a disk of the same radius r_0.

We have already discussed this problem analytically in Supplementary Note 2 and have found that the graph Laplacian Q is approximated by the Laplace-Beltrami operator up to corrections of order h^3, where $h = 0.496\,970$ for $\{3,7\}$, $h = 0.275\,798$ for $\{7,3\}$, and $h = 0.165\,657$ for $6.6.7$. We therefore anticipate these correction to be smallest for the $6.6.7$ tessellation, in agreement with the area per vertex being smallest for this tessellation. We now verify this explicitly for the three tessellations on disks of radius $r_0 = 0.99$ by numerically computing the spectrum (eigenvalue as a function of angular momentum) and comparing the dispersion to the one obtained from the continuum, as we did in Fig. **2** in the main text. Note that because of finite-size effects, our method fails to correctly identify the angular momentum of certain highly excited states (see the corresponding discussion in the Methods section). The results are shown in Supplementary Figure **2** and we observe that the difference between lattice (orange diamonds) and continuum (blue disks) dispersion is smaller for tessellations with small area per site, i.e., when the total number of sites is larger.

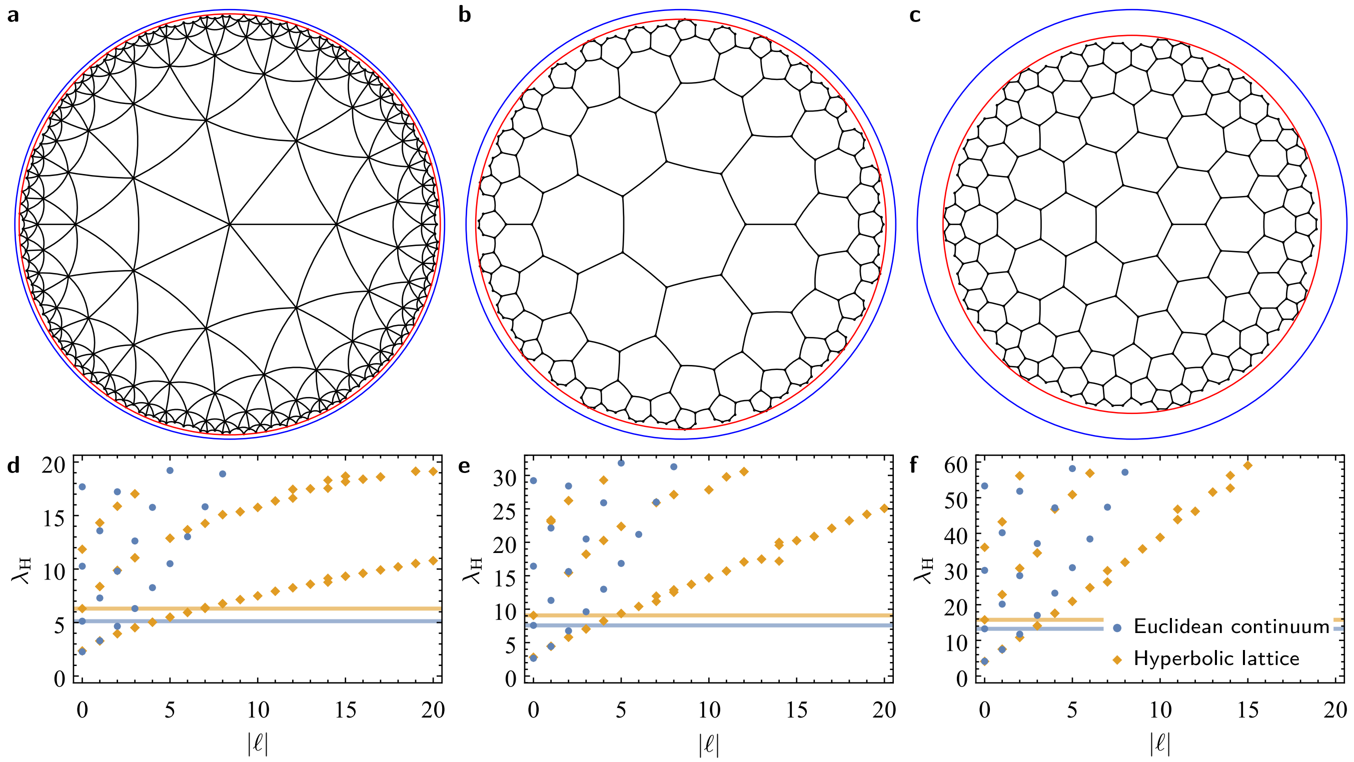

Supplementary Figure 3. **Comparison of hyperbolic tessellations for fixed number of vertices. a,b,c** Tessellations $\{3,7\}$, $\{7,3\}$ and $6.6.7$ of the hyperbolic plane in Poincaré disk representation (the unit circle is shown in blue), respectively, with approximately 275 vertices (the exact numbers of vertices are 274, 273 and 280, respectively). The red circle indicates the bounding circle of each tessellation with radii $r_0 = 0.98$, 0.955 and 0.88, respectively. **d,e,f** Angular momentum dispersion of the eigenstates of the graph Laplacian for the three tessellations (orange diamonds) compared to the same for the eigenmodes of the continuum Laplace operator on the Euclidean drum with the same radius r_0 (blue disks). Horizontal lines of the corresponding color indicate the eigenvalue of the $(n,\ell) = (1,0)$ mode in each geometry. The difference in the number of modes below the orange and the blue line quantifies the spectral reordering between the hyperbolic and the Euclidean disk. Note that the eigenvalues λ_{E} of the Euclidean drum are rescaled and shifted to allow for a better qualitative comparison to the hyperbolic dispersion, i.e., to emphasize the reordering of eigenstates.

b. Signatures of negative curvature

Above we have answered the question which of the three tessellations gives the best approximation of the continuum for a disk with fixed radius $r_0 < 1$. Experimentally, however, we are interested in a different question: For a given number of vertices (sites), which tessellation gives the strongest signatures of negative curvature? Naturally, we expect tessellations that cover a larger area of the Poincaré disk to exhibit stronger signatures of the negative curvature. Therefore, a large area per vertex is desirable. According to Supplementary Equation (30) and the values given in the paragraph following that equation, the $\{3,7\}$ tessellation is the one with the largest area per vertex out of the three under consideration.

We fix the (approximate) number of sites to 275 and construct the tessellations such that they consist of full shells. The resulting lattices are shown in Supplementary Figure 3. Here, we compare the angular momentum dispersion to the dispersion obtained from the eigenmodes of the continuum Laplace-Beltrami operator on the Euclidean drum of the same radius r_0, each. The signature of negative curvature which we have identified in the main text, i.e., the reordering of the eigenstates compared to the Euclidean case, is with a difference of four states strongest for the $\{3,7\}$ tessellation (panels **a, d**) and reduced to only a single state for 6.6.7 (panels **c, f**). Therefore, to reveal the spectral reordering in an experimental realization with a limited number of sites, it may be desirable to opt for the $\{3,7\}$ tessellation.

c. Role of rotation symmetry

Finally, we discuss the role of rotation symmetry. The tessellations shown in Supplementary Figure 2b,c can be shifted such that they have a vertex at the centre of the disk. As we argued in the main text, this is advantageous in order to to excite and detect $\ell = 0$ modes which have a maximum amplitude at the centre of the disk. However, in the case of the $\{7,3\}$ tessellation, the seven-fold rotation symmetry is broken down to a three-fold rotation symmetry, while in the case of the 6.6.7 tessellation no

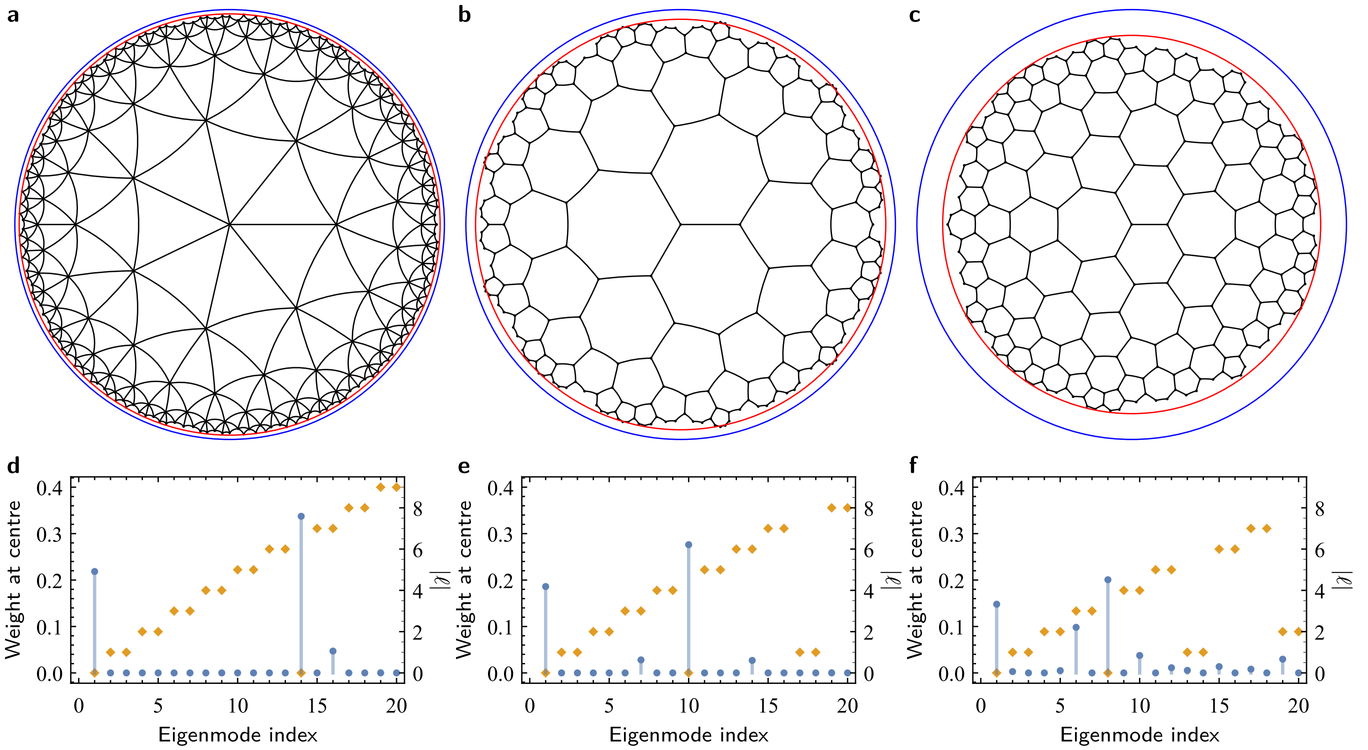

Supplementary Figure 4. **Comparison of hyperbolic tessellations with different order of rotation symmetry. a,b,c** Tessellations $\{3, 7\}$, $\{7, 3\}$ and 6.6.7 of the hyperbolic plane in Poincaré disk representation (the unit circle is shown in blue), respectively, with approximately 275 vertices (the exact numbers of vertices are 274, 271 and 271, respectively) and a vertex at the centre. The red circle indicates the bounding circle of each tessellation with radii $r_0 = 0.98$, 0.96 and 0.88, respectively. **d,e,f** For the first 20 eigenmodes (counting degenerate modes), the absolute value of their weight at the central vertex (blue disks, left vertical axis) and absolute value of their angular momentum $|\ell|$ (orange diamonds, right vertical axis) are shown.

rotation symmetry is remaining at all. These shifted tessellations are displayed in Supplementary Figure 4b,c.

In Supplementary Figure 4d–f we show for each of the first 20 eigenmodes of the graph Laplacian of each considered tessellation their angular momentum ℓ and their weight at the central vertex. From the continuum we expect that only $\ell = 0$ modes have non-vanishing weight at that vertex. This, indeed, holds on the $\{3, 7\}$ lattice for all $|\ell| \leq 6$. After that, we observe that eigenvalues of the two $|\ell| = 7$ modes (and similarly for integer multiples of 7) are split (cf. Supplementary Figure 2d), in stark contrast with the continuum case where such modes are degenerate. We also observe that one of these two modes acquires a non-zero weight at the central vertex. An analogous feature is observed for the $\{7, 3\}$ tessellation, where the modes with $|\ell|$ being integer multiples of 3 are similarly contaminated. Finally, the situation for the 6.6.7 tessellation is even less ideal as here most of the modes acquire a non-vanishing weight at the central vertex.

Therefore, we conclude that a small order of rotation symmetry leads to a larger number of $\ell \neq 0$ eigenmodes with non-vanishing weight at the central vertex. This in turn prevents us from easily detecting (and exciting) $\ell = 0$ modes via the central vertex, as stated in the main text.

SUPPLEMENTARY NOTE 4. PARASITIC RESISTANCES

Resolving individual peaks in the resonance spectrum requires a sufficiently high Q factor for all circuit elements. With increasing parasitic resistances, the resonance peaks of individual modes widen and flatten, making them harder to identify in an impedance sweep. In practice, inductors are the main source of parasitic resistances in our circuit. The Q factor of an inductor is defined through its impedance as $Q(\omega) = \frac{|Z_L(\omega)|}{\mathrm{Re}\{Z_L(\omega)\}}$. For $Q \gg 1$, we approximate $|Z_L| \approx |\mathrm{i}\omega L|$, and obtain $Z_L = \omega(\mathrm{i} + 1/Q)L$. Supplementary Figure 5 compares simulated impedance sweeps of node 18 for several constant Q factors of the inductors and the measured values. For Q factors of 50 and 20, all relevant impedance peaks can be easily identified, while at a Q factor of 10, the Peak at 0.906 MHz is no longer recognizable.

Supplementary Figure 5 shows that the measured data is consistent with $Q > 50$ in the measured frequency interval. For a

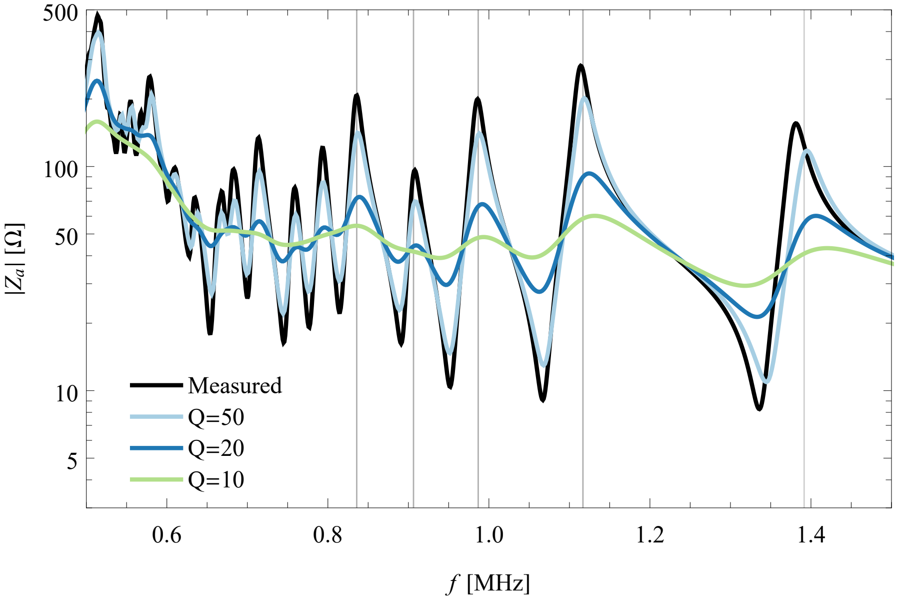

Supplementary Figure 5. Comparison of the simulated impedance spectrum at node 18 for different Q factors of the inductors and measurement values.

given eigenvector V_n of the hopping matrix M with corresponding eigenvalue λ_n, the Laplacian equation

$$\left(\mathrm{i}\,\omega\,C\,M + \frac{1}{\omega(\mathrm{i}+1/Q)L}\mathbb{1}\right)V_n = 0 \tag{31}$$

reduces to the scalar equation

$$\mathrm{i}\omega C\lambda_n + \frac{1}{\omega(\mathrm{i}+1/Q)L} = 0, \tag{32}$$

which is equivalent to that of a simple serial R-L-C oscillator. Note that in this equation, M denotes the hopping matrix, since Q is already used for the Q-factor. Since real and imaginary part of a serial oscillator's eigenfrequency are related by $\mathrm{Re}\{\omega_0\}/\,\mathrm{Im}\{\omega_0\} \approx 2Q$, we expect decay times of free oscillations in the circuit network to exceed 100 oscillation periods.

SUPPLEMENTARY NOTE 5. EXTENDED ANALYSIS OF MEASURED EIGENMODES

In this section we discuss additional data on the measured eigenmodes and perform an extended comparison to theory. We present extended versions of the right panel of Fig. **2**b and Fig. **3**c in the main text, and we quantitatively analyze the deviations of the experimentally extracted data from the theoretical prediction based on the Laplacian matrix of the hyperbolic lattice.

To compare the experimental results to theoretical predictions, the first step is to match the eigenmodes. In the main text, we have used a simple Fourier transform on the outermost sites to determine the angular momentum ℓ and match the modes according to the quantum numbers n, ℓ. As discussed at that point, such a procedure works well for the lowest couple of eigenmodes, but becomes increasingly inaccurate with increasing n and ℓ. To analyze more modes, we therefore use the alternative method described in Supplementary Note 3 a. Recall that there we theoretically analyzed the overlap of the graph Laplacian's eigenvectors to the discretized eigenmodes of the continuum Laplace-Beltrami operator (on the appropriate disk and with appropriate boundary conditions). We repeat here an analogous analysis for the experimentally extracted mode profiles (see Supplementary Figure 6 for several examples) and determine $(n, |\ell|)$ by identifying the continuous eigenmode for which the overlap is largest (see Supplementary Figure 7 for the results).

In Supplementary Figure 7 we further compare the 16 eigenmodes that we were able to excite, measure, and identify successfully with the corresponding eigenvectors of the adjacency matrix obtained numerically from the theoretical hyperbolic lattice. The deviation from theory is quantified by (1) the point-wise difference and by (2) the overlap. The former is plotted in Supplementary Figure 7 and analyzed in Supplementary Figure 8b, which shows the mean and standard deviation of the point-wise difference between experiment and theory (green squares). Supplementary Figure 8b also shows the overlap of the experimentally measured eigenmodes with the theoretical predictions (red triangles). Furthermore, we compare the measured and predicted eigenvalues

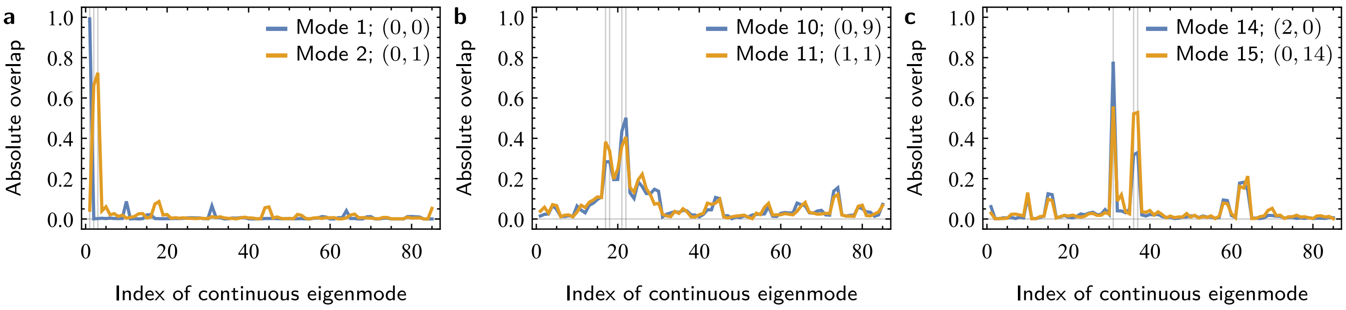

Supplementary Figure 6. **Identifying the quantum numbers of the measured eigenmodes.** Absolute value of the overlap of some of the measured modes with the eigenmodes of the Laplace-Beltrami operator with Dirichlet boundary conditions on a disk of radius $r_0 = 0.94$. The vertical gray lines indicate the positions of the maxima of the overlap, which allow us to assign quantum numbers $(n, |\ell|)$ to each mode (see legend). **a** The low-energy modes (corresponding to small λ) can be easily matched to continuous eigenmodes because they only show significant overlap with a single mode with $\ell = 0$ (e.g., mode 1; blue line) or a pair of modes with $\pm\ell$ (e.g., mode 2; orange line). For some specific modes, this is not the case: **b** Modes 10 and 11 both have significant overlap with $(n, |\ell|) = (0, 9)$ and $(1, 1)$, which can be understood from the fact that these two modes are close to being accidentally degenerate (Supplementary Figure 8a). **c** The breaking of the continuous rotation symmetry by the lattice lifts the degeneracy of the $(1, -14)$ and $(1, +14)$ mode such that one of them ends up close in eigenvalue to the $(2, 0)$ mode, leading to their hybridization and to a significant overlap with the corresponding continuous eigenmodes.

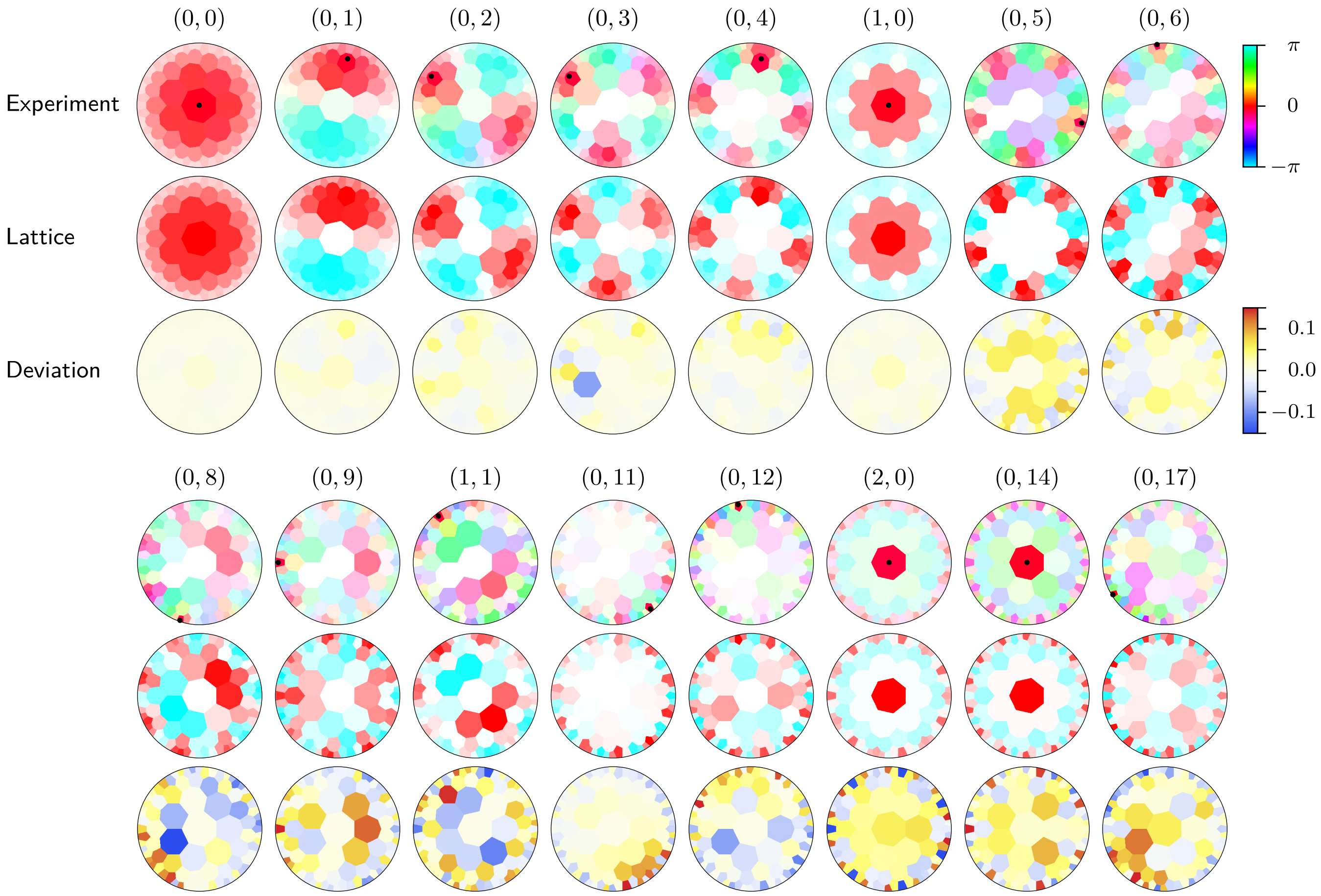

Supplementary Figure 7. **Comparison of measured eigenmodes to eigenvectors of the graph Laplacian matrix.** The three rows (continued in the lower half of the figure) show the following data. **Experiment**: the voltage profile of the measured eigenmodes with saturation encoding the magnitude as a fraction of the voltage (white denotes 0 and full saturation 1) at the input node (black dots) and color encoding the phase relative to the reference voltage (see legend on the right). **Lattice**: eigenvectors obtained from diagonalizing the Laplacian matrix defined by hyperbolic lattice (saturation and color as for the experiment). **Deviation**: difference between the normalized experimental data and the data on the lattice (see legend on the right). For each mode the quantum numbers n and $|\ell|$ are extracted by determining the continuous eigenmode with maximal overlap (see Supplementary Figure 6) and given at the top in the format $(n, |\ell|)$. The modes from experiment and theory are matched according to those numbers.

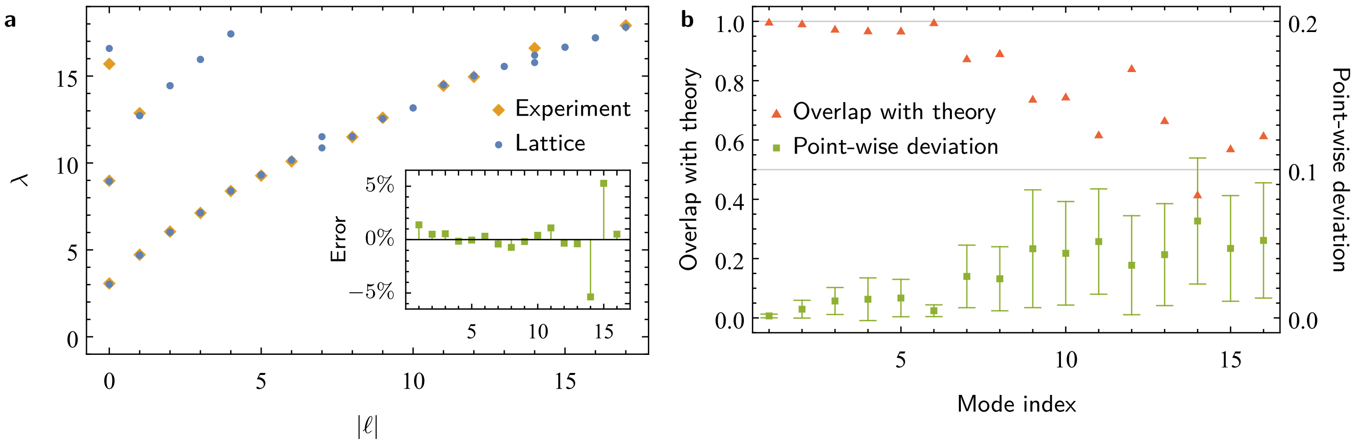

Supplementary Figure 8. Quantitative comparison measured eigenmodes and eigenvalues to theory. a Angular momentum dispersion, i.e., eigenvalue λ vs. the absolute value of the angular momentum ℓ for each eigenmode. Data obtained from measurements of the electric circuit (orange diamonds) and from diagonalizing the Laplacian matrix defined by the hyperbolic lattice (blue disks) are shown. The inset shows the relative error in the experimental data compared to the theoretical prediction (horizontal axis: mode index according to increasing λ in the experimental data); there are only two outliers (modes 14 and 15) with a relative error significantly larger than 1% (see text). **b** Comparison of the experimentally extracted eigenmodes to the eigenvector of the Laplacian matrix (both shown in Supplementary Figure 7). The red triangles show the absolute overlap of the experimentally and theoretically obtained eigenvectors and the green squares the mean of the absolute value of the point-wise deviation (interval marks indicate the standard deviation computed over all the nodes in the circuit). Again, mode 14 can be identified as an outlier (see text).

in Supplementary Figure 8a (an extended version of the right panel of Fig. **2**b in the main text), i.e., the eigenvalue as a function of $|\ell|$ for both the experimental as well as theoretical (lattice) data with relative errors shown in the inset.

We observe that the relative error for almost all of the 16 eigenmodes is below 2% (cf. Supplementary Figure 8a); the two outliers, modes 14 and 15, are discussed below. In contrast, Supplementary Figure 8b shows that the measured eigenmodes agree very well with theory for modes 1 to 6 after which the deviations start to increase. This is reflected both in the overlap of the measured modes with the theoretically expected ones, as well as in the point-wise deviations at the individual circuit nodes (plotted also in Supplementary Figure 7). Note that additionally, due to parasitic effects, the experimental data shows increasing deviations in the phase compared to the theory where only $0, \pi$ phases occur (cf. Supplementary Figure 7).

The deviations in λ, i.e., in the eigenfrequencies of the circuit, are weakly dependent on the index of the excited modes (except for the outliers mentioned above). This indicates that these deviations most likely can be attributed to parasitic effects and to disorder in the circuit components, as these are both expected to exhibit such a weak dependence on the index of the excited modes. The eigenfrequencies are extracted from impedance measurements such as Fig. **3**b in the main text; as long as the peaks are well separated, they can be accurately measured. Note that problems can arise when two modes are close to each other in eigenvalue, i.e., almost accidentally degenerate, which is the case for modes 10 and 11. Supplementary Figure 6b shows that both modes have significant overlap with the $(n, |\ell|) = (0, 9)$ and $(1, 1)$ eigenmodes of the continuum Laplace-Beltrami operator. This makes it more challenging to excite and assign quantum numbers to those modes. On the other hand, the mode profiles are much more sensitive to other error sources. First, in the experiment, it is impossible to excite exactly a single mode, generally a superposition of several modes is excited. When the eigenmodes are well separated in frequency or if the input node lies in a nodal plane of many other eigenmodes, the additional eigenmodes have a small weight in the superposition. However, with increasing mode number the frequency separation is reduced, such that the deviations from theory increase gradually.

Finally, we comment on the missing data for the $|\ell| = 7$ mode as well as the large difference of the experimentally extracted and theoretically predicted eigenvalues for the $(n, |\ell|) = (2, 0)$ and $(1, 14)$ modes (modes 14 and 15 have a relative error that is significantly larger than the typical error, cf. Supplementary Figure 8a). As discussed in Supplementary Note 3 c, for rotation symmetry of finite order some modes with $\ell \neq 0$ attain a non-vanishing amplitude at the origin and the degeneracy with the second mode with identical $n, |\ell|$ is lifted (only one of the two modes has a significant amplitude at the origin). We have not managed to cleanly excite the $(1, 7)$ modes, but the described phenomenon is visible in the data for the $(1, 14)$ mode (cf. Supplementary Figure 7). This also allows us to understand the large deviation of the eigenvalues of the $(2, 0)$ and $(1, 14)$ modes: both have significant weight at the origin (cf. Supplementary Figure 7) and eigenvalues that are expected to be very close to each other (cf. Supplementary Figure 8a). Therefore, it is difficult to excite only one of them, such that the experimentally excited modes are very likely superpositions of the two. This is reflected in the overlap of the measured modes with the eigenmodes of the continuum Laplace-Beltrami operator in Supplementary Figure 6c. While based on the voltage profile (either visually or via the Fourier transform) the lower mode is identified as the $\ell = 0$ mode, the eigenvalues would suggest the opposite (cf. Supplementary Figure 8a). Choosing different input nodes should resolve this issue.

In conclusion, we find that getting accurate data on the eigenvalues is not a problem as long as the modes can be cleanly excited, while the error in the eigenmode profiles increases gradually with increasing mode number. Nevertheless, correctly identifying the modes remains possible even with reduced accuracy of the mode profiles. To recognize the reordering of the eigenmode compared to flat space, the accuracy of our experimental setup is more than sufficient: only the first six modes are required, for which the overlap with theory is above 95%; but we have demonstrated that, already without any additional optimization, it is possible to find good agreement with theory for higher modes as well.

SUPPLEMENTARY NOTE 6. SIGNAL PROPAGATION IN THE ELECTRIC CIRCUIT NETWORK

In this section we briefly explain the time-dependent behavior of the hyperbolic circles of constant phases in Supplementary Movie 1. While we have already discussed the results at fixed times in the main text, the time-dependence requires some additional explanation. In particular, we observe that the hyperbolic circles of constant phases are falling into the input node. This is a consequence of our specific electric circuit network being a negative-index metamaterial (also called left-handed), i.e., having a negative refractive index.

The propagation of waves on a drum is generally given by the following differential equation involving the Laplace-Beltrami operator:

$$\frac{1}{c^2}\frac{\partial^2}{\partial t^2}u(t,x,y) - \Delta_{\mathrm{g}}u(t,x,y) = 0 \tag{33}$$

with the wave speed c determined by the medium. In the Euclidean case, for example, the wave-equation leads to the dispersion $\omega(k) = c\,|\mathbf{k}|$ with the two-dimensional momentum vector $\mathbf{k}$; thus, phase and group velocity are equal: $v_p = v_g = c$. The situation in the experiment corresponds to an additional inhomogenous source term $S(x,y,t)\sin(\omega t)$, where S is localized both in space (where the excitation happens) and in time (pulse-like), i.e.,

$$\frac{1}{c^2}\frac{\partial^2}{\partial t^2}u(t,x,y) - \Delta_{\mathrm{g}}u(t,x,y) = S(x,y,t)\sin(\omega t). \tag{34}$$

The source term leads to an excitation of eigenmodes of the drum, i.e., of $-\Delta_{\mathrm{g}}$, according to its frequency spectrum and the pulse propagates across the drum with speed c.

For an electric circuit there are some important differences to the dynamics, even though the situation is conceptually the same. According to Kirchhoff's law, the differential equation governing our electric circuit network is

$$\frac{\partial}{\partial t}I_a = CQ_{ab}\frac{\partial^2}{\partial t^2}V_b - \frac{1}{L}V_a, \tag{35}$$

where $I_a(t)$ and $V_a(t)$ are the input current and voltage at node a, C the capacitance coupling two adjacent nodes, L the inductance to ground for each node and Q_{ab} the graph Laplacian describing the (capacitive) connections between the nodes. The continuum limit therefore is

$$C\frac{\partial^2}{\partial t^2}\Delta_{\mathrm{g}}V(t,x,y) - \frac{1}{L}V(t,x,y) = \frac{\partial}{\partial t}I(t,x,y) \tag{36}$$

where the voltage field $V(t,x,y)$ takes the role of $u(t,x,y)$ and the time-derivative of the input current $\frac{\partial}{\partial t}I(t,x,y)$ the role of the source.

The modified wave-equation

$$LC\frac{\partial^2}{\partial t^2}\Delta_{\mathrm{g}}u(t,x,y) - u(t,x,y) = 0 \tag{37}$$

results in the group velocity v_g having the opposite sign compared to the phase velocity v_p. In the Euclidean case, for example, the dispersion is

$$\omega(k) = \frac{1}{\sqrt{LC}}\frac{1}{k}, \tag{38}$$

which implies

$$v_g = \frac{\mathrm{d}\omega}{\mathrm{d}k} = -\frac{1}{\sqrt{LC}}\frac{1}{k^2} = -v_p. \tag{39}$$

This explains the observation in Supplementary Movie 1 that the hyperbolic circles of constant phase seem to fall into the input node (with v_p), while the excited voltage pulse is propagating away from it (with v_g).

---

* These two authors contributed equally to this work.
† Correspondence to titus.neupert@uzh.ch, rthomale@physik.uni-wuerzburg.de. and tomas.bzdusek@psi.ch.

[1] Boettcher, I., Bienias, P., Belyansky, R., Kollár, A. J. & Gorshkov, A. V. Quantum simulation of hyperbolic space with circuit quantum electrodynamics: From graphs to geometry. *Phys. Rev. A* **102**, 032208 (2020).
