# Supplementary material for: Simulating hyperbolic space on a circuit board
Source: Nat Commun. 2022 Jul 28;13:4373. doi: 10.1038/s41467-022-32042-4 (PMC9334621; doi:10.1038/s41467-022-32042-4)
Supplement: Supplementary file 3 — Description of Additional Supplementary Files [file 41467_2022_32042_MOESM3_ESM.docx]

**Description of Additional Supplementary Files**

**Supplementary Movie 1: Measured signal propagation in the electric circuit.** The application of a short and spatially localized pulse applied to node 31 (blue curve in the left panel) leads to a wave propagating through the circuit. The voltage response at node 31 is shown as an orange curve in the left panel and the instantaneous phase at each node in the right panel. The nodes are indicated by black dots, and concentric hyperbolic circles with centre at node 31 are shown in black to illustrate the hyperbolic metric.
